# Label Accuracy of Legal Oral Cannabis Oil Products in Ontario, Canada

**DOI:** 10.1001/jamanetworkopen.2024.14922

**Published:** 2024-06-05

**Authors:** Amanda Doggett, Allan Fein, Tracey Campbell, Nicola Henriquez, Jason W. Busse, James MacKillop

**Affiliations:** 1 Michael G. DeGroote Centre for Medicinal Cannabis Research, McMaster University and St Joseph’s Healthcare Hamilton, Hamilton, Ontario, Canada; 2 Peter Boris Centre for Addictions Research, McMaster University and St Joseph’s Healthcare Hamilton, Hamilton, Ontario, Canada; 3 Department of Psychiatry and Behavioural Neurosciences, McMaster University, Hamilton, Ontario, Canada; 4 Centre for Microbial Chemical Biology, McMaster University, Hamilton, Ontario, Canada; 5 Department of Anesthesia, McMaster University, Hamilton, Ontario, Canada; 6 Department of Health Research Methods, Evidence, and Impact, McMaster University, Hamilton, Ontario, Canada

## Abstract

This case series compares amounts of tetrahydrocannabinol and cannabidiol reported on product labels vs levels found in laboratory testing in legal oral cannabis oil products in Ontario, Canada.

## Introduction

In October 2018, Canada legalized cannabis for nonmedical (recreational) use. One component of the federal system was quality control, including cannabis labeling requirements that specify the allowable variance between labeled and actual amounts of tetrahydrocannabinol (THC) and cannabidiol (CBD) in a commercial product.^1^ Research examining legal cannabis products has found high rates of label inaccuracy for THC and CBD,^2,3^ but to our knowledge, no study has examined label accuracy of cannabis products in the legal Canadian market.

## Methods

Between November 2021 and January 2022, this case series study tracked all oral oil products available on the Ontario Cannabis Store (OCS) website and randomly selected 30 products that were available at least twice during the study period. Amounts of CBD and THC in each product were quantified using high-performance liquid chromatography at the Centre for Microbial Chemical Biology at McMaster University (Health Canada analytics license: LIC-BZJ23VHQ0X-2021). Federal cannabis regulations indicate that the allowable variability for extracts is 15% above or below the product’s labeled amount.^1^ For comparability to other research, we summed the number of products that exceeded the variability limit and divided by the number of products tested. Given that very low–concentration products could exceed this threshold with trivial absolute increases, we conducted a subgroup analysis of higher-potency products (≥2.5 mg/g THC) as defined by OCS consumer guidance.^4^ See eMethods in Supplement 1 for detailed methodology. The DeGroote Centre for Medicinal Cannabis Research determined that ethical review was not required for this study given that it involved no human participants or animal subjects.

## Results

**Figure 1.**
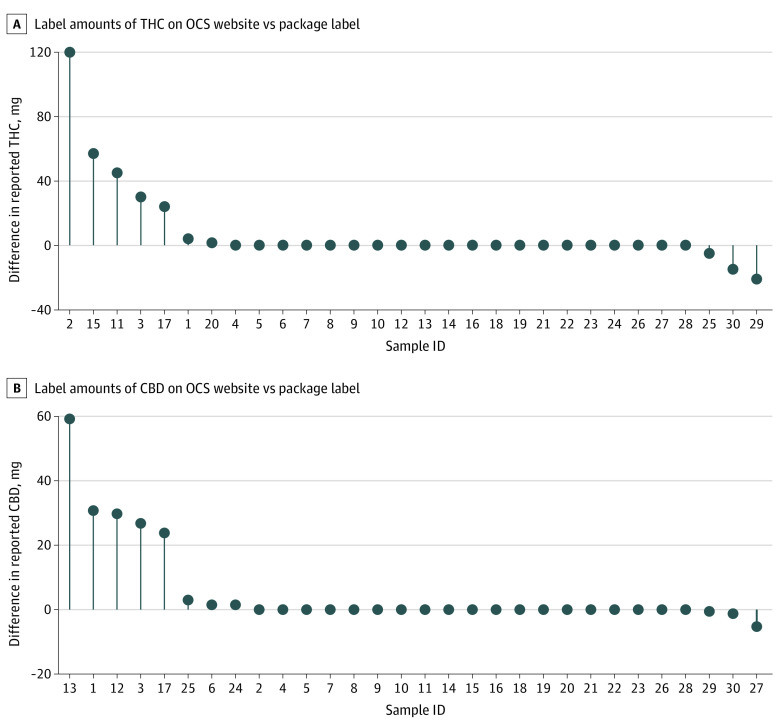
Amounts in Product Labeling vs Website Differences are shown between overall tetrahydrocannabinol (THC) and cannabidiol (CBD) in products indicated on package labeling and amounts indicated on the Ontario Cannabis Store (OCS) website at the time of purchase. Lines indicate the amount listed on the OSC website at the time of purchase subtracted from the amount on the physical package label of the product received in milligram units (ie, total milligrams of THC or CBD in the bottle). In other words, positive differences reflect greater THC or CBD content on the physical label compared with amounts on the OCS website, and negative differences indicate lesser amounts. Most products (26 products for THC and 28 products for CBD) listed ranged values on the OCS website. Calculated differences from label values represent a conservative approach, as discrepancies from the highest end of the range (for cases with positive differences) or lowest end of the range (for cases with negative differences).

There were discrepancies between information on the OCS website regarding advertised amounts of THC and CBD and physical product labels for 10 of 30 oil products (33.3%). (Figure 1). We also found internal inconsistency, with 5 products (16.7%) labeled with discordant *THC/CBD* (denotes active cannabinoid content) and *total THC/CBD* (denotes cannabinoid content after product is heated for consumption) concentrations, which should be identical for extract type products.^5^ The product with the largest discrepancy was labeled as having 5 mg/g CBD but 26 mg/g total CBD.

**Figure 2.**
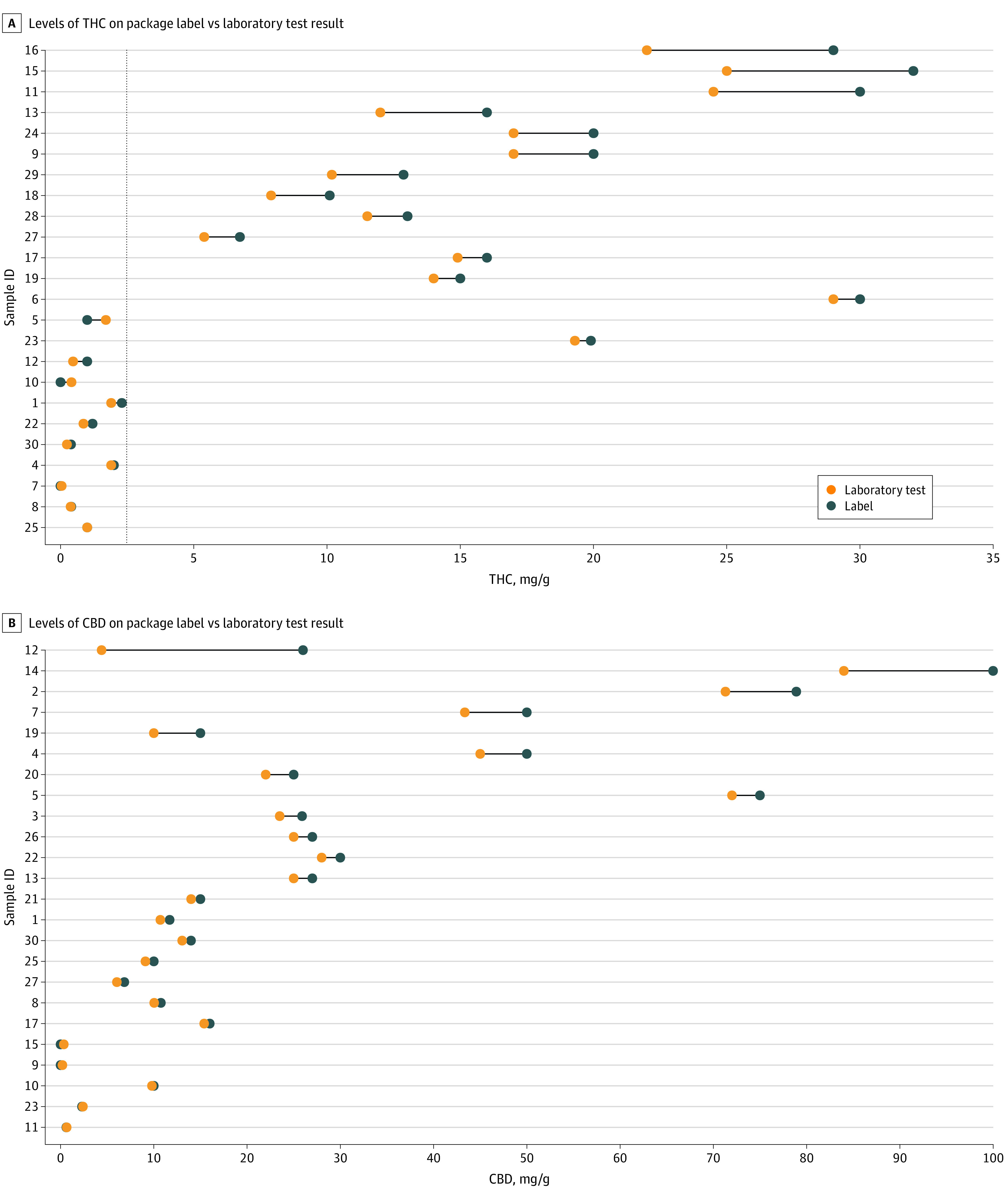
Label Amounts vs Laboratory Testing Some samples are not displayed due to a zero absolute difference value between labels and testing (samples 6, 16, 18, 24, 28, and 29 for cannabidiol [CBD] and samples 2, 3, 14, 20, 21, and 26 for tetrahydrocannabinol [THC]). The dashed line indicates 2.5 mg/g.

Figure 2 shows differences between THC and CBD amounts on product labels and product amounts by laboratory assay. Overall, 12 products (40.0%) were outside the variability limit for THC and 3 products (10.0%) were outside the variability limit for CBD (due to greater labeled vs laboratory-tested amounts for all but 1 product). Among 16 products that had a label amount of 2.5 mg/g THC or greater, 7 products (43.8%) had amounts that were lower than what was labeled by more than 15%.

## Discussion

To our knowledge, this case series study is the first study of label accuracy of cannabis products in the legal Canadian market. We found discrepancies at multiple levels. One-third of purchased products differed from their online THC and CBD descriptions, and 16.7% had conflicting information on the physical label. Compared with assay levels, amounts for 40.0% of products were outside variability limits for THC and 10.0% for CBD; all but 1 were instances of overlabeling. Among higher-potency products (≥2.5 mg/g THC), nearly half were incorrectly labeled as containing THC amounts that were greater than what was indicated by laboratory testing by more than 15%.

Our findings suggest that inaccurate labeling of cannabis oil products in the legal Canadian market is common, with most discrepancies due to labeling products with greater THC or CBD content than was present. No products contained more THC than labeled at an amount that would be expected to have substantively different psychoactive effects. However, given that many medical consumers obtain products from the nonmedical market,^6^ one implication is inaccurate dosing. Altogether, these findings suggest a need for greater quality control in the Canadian legal cannabis market and undermine the assumption that a legal market is an assurance of accurate labeling. Limitations of our study include a focus on cannabis oils, with uncertain generalizability to other product types, and the sample size of 30 products, which is not representative of all available products in Ontario or Canadian markets.

## Notes

Supplement 1.
eMethods.

Supplement 2.
Data Sharing Statement
