# Supplementary material for: Label Accuracy of Legal Oral Cannabis Oil Products in Ontario, Canada
Source: JAMA Netw Open. 2024 Jun 5;7(6):e2414922. doi: 10.1001/jamanetworkopen.2024.14922 (PMC11154152; doi:10.1001/jamanetworkopen.2024.14922)
Supplement: Supplement 1. — eMethods. [file jamanetwopen-e2414922-s001.pdf]

Supplemental Online Content

Doggett A, Fein A, Campbell T, Henriquez N, Busse JW, MacKillop J. Label accuracy of legal oral cannabis oil products in Ontario, Canada. *JAMA Netw Open*. 2024;7(6):e2414922. doi:10.1001/jamanetworkopen.2024.14922

eMethods.

This supplemental material has been provided by the authors to give readers additional information about their work.

© 2024 Doggett A et al. *JAMA Network Open.*

Supplement I: Additional Methodological Details

Standards and Calibration Curves

Calibration curves for cannabinoids were linear over the range of interest with $r^2 > 0.99$. Calibration standards were prepared from individual commercially available certified reference standards at 1.0 mg/mL each in organic solvent. Equal volumes of reference standard were mixed and diluted with liquid chromatography mass spectrometry (LC/MS) grade acetonitrile to a concentration of 100.0 µg/mL. Serial dilutions were made to create the calibration curve at concentrations of 100.0, 50.0, 10.0, 5.0, 1.0, 0.5, and 0.1 µg/mL in acetonitrile. To prepare the calibration curve, the average area of the analyte response for each of the calibration standards injected was plotted against the concentration of the calibration standards. The calibration curves were used for accuracy and linearity determinations.

Sample Preparation

The Centre for Medicinal Cannabis Research purchased the cannabis products (Health Canada Institutional Research License: LIC-RMXR01YDD2-2021), and products were received directly from the Ontario Cannabis Store by The Centre for Microbial Chemical Biology (Health Canada Analytics License: LIC-BZJ23VHQ0X-2021) and were stored at room temperature in a secured location until time of processing. All products were analyzed prior to their listed expiry date. The solubility of the oil samples was tested in various solvents. The goal was to choose a solvent that solubilized the oil completely and was high performance liquid chromatography (HPLC) mobile phase compatible. The recovery of the cannabinoids was 100%, assuming perfect solubility of cannabinoids in the diluent solvent. Once the appropriate solvent was determined, the samples were processed in triplicate. A 100 µL aliquot of oil was added into a tared 10-mL volumetric

© 2024 Doggett A et al. *JAMA Network Open.*

flask. Approximately 8 mL of high purity LC/MS grade diluent solvent (acetonitrile, methanol or isopropanol) was added to the volumetric flask, capped and mixed by inversion. The volume was then brought up to 10 mL with the diluent solvent (100-fold sample dilution). An additional 10-fold dilution was performed by transferring a 100 µL aliquot of this solution into an amber glass 2-mL autosampler vial and adding 900 µL LC/MS grade acetonitrile. The samples were capped and vortexed briefly prior to injection on the HPLC (final dilution factor = 1000).

A volume of 5 µL was injected into an Agilent 1290 Infinity II HPLC with an Agilent 6495C iFunnel QQQ mass spectrometer for detection. Separation of the analytes was achieved on an Agilent RRHD Eclipse Plus C18 (100 mm x 2.1 mm, i.d., 1.8 µm) analytical column using mobile phases consisting of (A) 0.1% (v/v) formic acid in water and (B) acetonitrile and a constant flow of 0.2 mL/min. UV detection at 230 nm was used for quantification of the analytes. The typical retention times for CBD and THC were approximately 6.9 and 9.7 minutes, respectively. The same conditions were used for all injections and the retention times were consistent throughout each run and between batches.

Calculations

According to the Cannabis Regulations, cannabis oils (or a non-solid containing cannabis, as they are defined in the regulations) are classified as extracts, and Cannabis Regulation Section 97(1) states that extracts must not contain less than 85% or more than 115% of quantity or concentration of THC or CBD listed on the label (i.e., ±15%). However, Section 97.1(1) indicates that the variability limit is ± 25% if "*a cannabis product that is not in discrete units is represented as being able to be divided into discrete units*" which can also apply to cannabis oils. Currently, it is not explicitly stated in the regulations which variability limit should apply to

cannabis oils, but since the former is more specific than the latter, ±15% was used as the primary definition.

At the time of purchase, THC and CBD concentrations were recorded from the OCS website in total milligrams. In order to compare package labels to the website values, total THC/CBD was multiplied by the container size (in mL); a 1:1 calculation was used because it aligns with how products on the OCS website convert between mg and mg/g for cannabis oils. If the OCS website listed a THC or CBD range, differences from the label were calculated by subtracting from the upper limit of the range (if the label amount was greater) or the lower limit of the range (if the label amount was lesser). To examine differences between product labels and actual amounts determined by laboratory assay, total THC and total CBD amounts in mg/g from the product labels were used.

© 2024 Doggett A et al. *JAMA Network Open.*
