# Supplementary material for: Label Accuracy of Legal Oral Cannabis Oil Products in Ontario, Canada
Source: JAMA Netw Open. 2024 Jun 5;7(6):e2414922. doi: 10.1001/jamanetworkopen.2024.14922 (PMC11154152; doi:10.1001/jamanetworkopen.2024.14922)
Supplement: Supplement 2. — Data Sharing Statement [file jamanetwopen-e2414922-s002.pdf]

Data Sharing Statement

Doggett. Label Accuracy of Legal Oral Cannabis Oil Products in Ontario, Canada. *JAMA Netw Open*. Published online June 5, 2024. doi:10.1001/jamanetworkopen.2024.14922

Data
Data available: No
